# Diagnostic features of paediatric catatonia: multisite retrospective cohort study

**DOI:** 10.1192/bjo.2024.61

**Published:** 2024-04-30

**Authors:** Joshua R. Smith, Tasia York, Isaac Baldwin, Catherine Fuchs, Gregory Fricchione, James Luccarelli

**Affiliations:** Division of Child and Adolescent Psychiatry, Department of Psychiatry and Behavioral Sciences, Vanderbilt University Medical Center at Village of Vanderbilt, Tennessee, USA; and Vanderbilt Kennedy Center, Vanderbilt University, Tennessee, USA; Division of Child and Adolescent Psychiatry, Department of Psychiatry and Behavioral Sciences, Vanderbilt University Medical Center at Village of Vanderbilt, Tennessee, USA; Division of General Psychiatry, Department of Psychiatry and Behavioral Sciences, Vanderbilt University Medical Center, Tennessee, USA; Harvard Medical School, Massachusetts, USA; and Department of Psychiatry, Massachusetts General Hospital, Massachusetts, USA

**Keywords:** Neurodevelopmental disorders, childhood experience, comorbidity, epidemiology, patients

## Abstract

**Background:**

Catatonia is a neuropsychiatric disorder characterised by psychomotor changes that can affect individuals across the lifespan. Although features of catatonia have been described in adults, the most common clinical symptoms among paediatric patients with catatonia are not well characterised.

**Aims:**

The goal of this study was to characterise the symptoms of catatonia demonstrated by paediatric patients, and to explore demographic and diagnostic factors associated with greater catatonia severity.

**Method:**

We conducted a multicentre retrospective cohort study, from 1 January 2018 to 6 January 2023, of patients aged 18 and younger with a clinical diagnosis of catatonia and symptom assessment using the Bush Francis Catatonia Rating Scale (BFCRS).

**Results:**

A total of 143 patients met inclusion criteria. The median age was 15 (interquartile range: 13–16) years and 66 (46.2%) patients were female. Neurodevelopmental disabilities were present in 55 (38.5%) patients. Patients demonstrated a mean of 6.0 ± 2.1 signs of catatonia on the Bush Francis Catatonia Screening Item, with a mean BFCRS score of 15.0 ± 5.9. Among the 23 items of the BFCRS, six were present in >50% of patients (staring, mutism, immobility/stupor, withdrawal, posturing/catalepsy, rigidity), and four were present in <20% of cases (waxy flexibility, mitgehen, gegenhalten, grasp reflex). In an adjusted model, patients with neurodevelopmental disorders demonstrated greater BFCRS severity than those with other diagnoses.

**Conclusions:**

Catatonia was diagnosed in a range of mental health conditions. Further research is needed to define optimal diagnostic criteria for catatonia in paediatric patients, and clarify the clinical course of the disorder.

Catatonia is a neuropsychiatric disorder categorised by changes in psychomotor function and affect. Although recognised as a clinical condition for nearly 150 years, the clinical understanding of catatonia has evolved from that of a subtype of schizophrenia to a condition with characteristic mental status and physical examination findings.^1^ The prevalence of catatonia across treatment settings has been estimated at 9.0%, based on a large meta-analysis of studies conducted largely in adults and largely within the psychiatric hospital setting.^2^ Numerous rating scales exist to measure the presence and severity of the clinical features of catatonia, which generally measure a relatively consistent set of findings,^3^ although there remain differences in measured signs. In particular, the DSM-5-TR lists 12 features of catatonia, of which 3 must be present for catatonia to be diagnosed. In contrast, the Bush Francis Catatonia Rating Scale (BFCRS),^4^ the most cited paper in the field of catatonia,^5^ consists of a 14-item Bush Francis Catatonia Screening Item (BFCSI) and a full 23-item BFCRS that measures a greater number of signs. The diagnostic structure and features of the BFCRS have been explored in a large cohort of 232 adults with catatonia,^6^ which found significant differences in the frequency of observed signs, from 74% with staring to 11% with combativeness.

Catatonia may also be present in paediatric patients,^7^ although it is diagnosed less often than in adults, with administrative claims records showing 900 paediatric patients diagnosed with catatonia in 2019 in the general hospital setting in the USA.^8,9^ Catatonia in paediatrics has been recognised as a particular comorbidity in individuals with a variety of medical conditions,^10,11^ psychotic disorders,^7,12^ neurodevelopmental disorders^13–15^ and genetic conditions.^16–18^ A timely diagnosis and intervention is critical, as paediatric catatonia is associated with significant morbidity and a 60-fold higher risk of death than the general population.^19^ Despite this remarkable clinical significance, research into paediatric catatonia has been limited by small sample sizes and generally single-centre studies, making it challenging to draw firm conclusions regarding the clinical features of paediatric catatonia and how they may differ from those of adults. This study presents an analysis of catatonic signs in a large, multicentre retrospective cohort of youth with a diagnosis of catatonia and clinical assessment using the BFCRS to describe the clinical features of paediatric catatonia. We compare clinical features in neurotypical individuals and in those with neurodevelopmental disorders, as well as features in patients with catatonia associated with medical diagnoses and non-medical diagnoses. Additionally, we explore demographic and diagnostic factors associated with greater catatonia severity, as measured by the BFCRS.

## Method

The clinical records of two large healthcare systems, one in North-Eastern USA and one in Southern USA, were queried for patients aged 18 years and younger with a discharge diagnosis of catatonia between 1 January 2018 and 6 January 2023.^20^ Patients were included in the study if they were aged 18 years or younger, had a clinical diagnosis of catatonia as confirmed in clinical documentation and had a full BFCRS documented at the time of initial catatonia diagnosis. The clinical diagnosis of catatonia required a minimum of two catatonic signs from the BFCSI. Primary discharge diagnosis (meaning the first non-catatonia diagnosis listed in the patient discharge summary), psychiatric comorbidities, demographics and BFCRS scores were then extracted from the clinical records. This study was approved by the institutional review board of each study site (Vanderbilt University approval number: 230097; Mass General Brigham approval number: 2022P000811), with a waiver of informed consent obtained from participants.

### Features of catatonia

Catatonic features were defined from the BFCRS.^4^ The first 14 items were graded on a binary scale as present or absent as part of the BFCSI. Overall catatonia severity was measured with the 23-item BFCRS. Of the items, six items are scored on a binary scale of 0 or 3, and 17 items are scored on a four-level ordinal scale from 0 to 3. The grading of the ordinal scale varies among items, with higher scores indicating more severe symptoms based on severity, duration or frequency of the individual item measured. Thus, the BFCRS has a maximum possible score of 69. The initial publication of the BFCRS noted an interrater reliability of 0.93 for the BFCRS total score, with a mean agreement of items of 88.2%. Interrater reliability for the BFCSI was reported as 0.95, with a mean agreement of items of 92.7%.^4^

### Statistical analysis

Demographics, BFCSI signs and BFCRS scores are presented with descriptive statistics, both in aggregate across all patients and for subgroups of patients with neurodevelopmental disorders and primary medical diagnoses associated with catatonia. Differences in median BFCSI symptoms were compared between patients with and without neurodevelopmental disorders, and with and without primary medical diagnoses, using the Mann–Whitney *U*-test given the non-normality of the data. In an exploratory statistical analysis, the total BFCRS score (dependent variable) was modelled with a generalised linear model, with age, gender, study site and primary diagnosis associated with catatonia (medical, psychotic disorder, mood disorder, neurodevelopmental disorder, unspecified catatonia) as independent variables, with all independent variables included in a single model. All analyses were done with SPSS version 29.0 for Windows.

## Results

### Patient demographics

Across both hospital systems, a total of 143 paediatric patients were diagnosed with catatonia and had BFCRS documentation at the time of initial catatonia diagnosis (Table 1). Of these, 66 (46.2%) were female. A total of 11 patients (7.7%) were younger than 9 years old, 22 patients (15.4%) were between 9 and 12 years old, 49 (34.4%) patients were between 13 and 15 years old, and 61 (42.7%) patients were between 16 and 18 years old; median age was 15 (interquartile range (IQR): 13–16) years, with a minimum age of 3 years. Catatonia was diagnosed in the in-patient setting for 131 (91.6%) patients, and in the out-patient setting for 12 (8.4%) patients. Baseline neurodevelopmental disorders were present in 55 (38.5%) patients diagnosed with catatonia. Of these, autism spectrum disorder (ASD) was diagnosed in 38 individuals (26.6%) and intellectual disability in 33 (23.1%) individuals, with 24 (16.8%) diagnosed with both conditions.

**Table 1:** Demographic characteristics of paediatric patients with a diagnosis of catatonia

Characteristic	*n*	%
Total number	143	
Gender		
Female	66	46.2
Male	77	53.8
Age, years, median (IQR)	15 (13–16)	
<9	11	7.7
9–12	22	15.4
13–15	49	34.3
16–18	61	42.7
Study site		
Site 1	107	74.8
Site 2	36	25.2
Treatment setting		
Out-patient	12	8.4
In-patient	131	91.6
Neurodevelopmental disorder (yes)	55	38.5
ASD without intellectual disability	14	9.8
ASD with intellectual disability	24	16.8
Intellectual disability without ASD	9	6.3
Other	8	5.6
Race		
Asian	9	6.3
Black	39	27.3
Native American	0	0
Pacific Islander	0	0
White	86	60.1
Other	5	3.5
Not reported	4	2.8
Ethnicity		
Hispanic	15	10.5
Not Hispanic	121	84.6
Not reported	7	4.9

IQR, interquartile range; ASD, autism spectrum disorder.

### Diagnoses associated with catatonia

Diagnostically, among paediatric patients with a diagnosis of catatonia, there was a range of associated medical and psychiatric diagnoses (Table 2). Psychotic disorders were the most frequently diagnosed primary disorder associated with catatonia (32.9%). Neurodevelopmental disorders were considered the primary diagnosis associated with catatonia in 26.6% of patients, and mood disorders were considered the diagnosis associated with catatonia in 14.7% of patients. Medical diagnoses were the primary associated diagnosis for 13.3% of patients, and 11.2% had a diagnosis of unspecified catatonia.

**Table 2:** Primary diagnosis for paediatric patients with a diagnosis of catatonia

Diagnosis	*n*	%
Psychotic disorders	47	32.9
Brief psychotic disorder	2	1.4
Schizophreniform disorder	3	2.1
Schizophrenia	11	7.7
Schizoaffective disorder	2	1.4
Unspecified psychosis	27	18.9
Substance-induced psychosis	2	1.4
Neurodevelopmental disorders	38	26.6
ASD without intellectual disability	8	5.6
ASD with intellectual disability	24	16.8
Intellectual disability without ASD	6	4.2
Mood disorders	21	14.7
Major depressive disorder	8	5.6
Bipolar disorder	11	7.7
Unspecified mood disorder	2	1.4
Medical diagnoses	19	13.3
Autoimmune encephalitis	13	9.1
Delirium	3	2.1
Systemic lupus erythematosus	2	1.4
Wilson's disease	1	0.7
Unspecified catatonia	16	11.2
Post-traumatic stress disorder	2	1.4

ASD, autism spectrum disorder.

### Catatonic signs

**Fig. 1:**
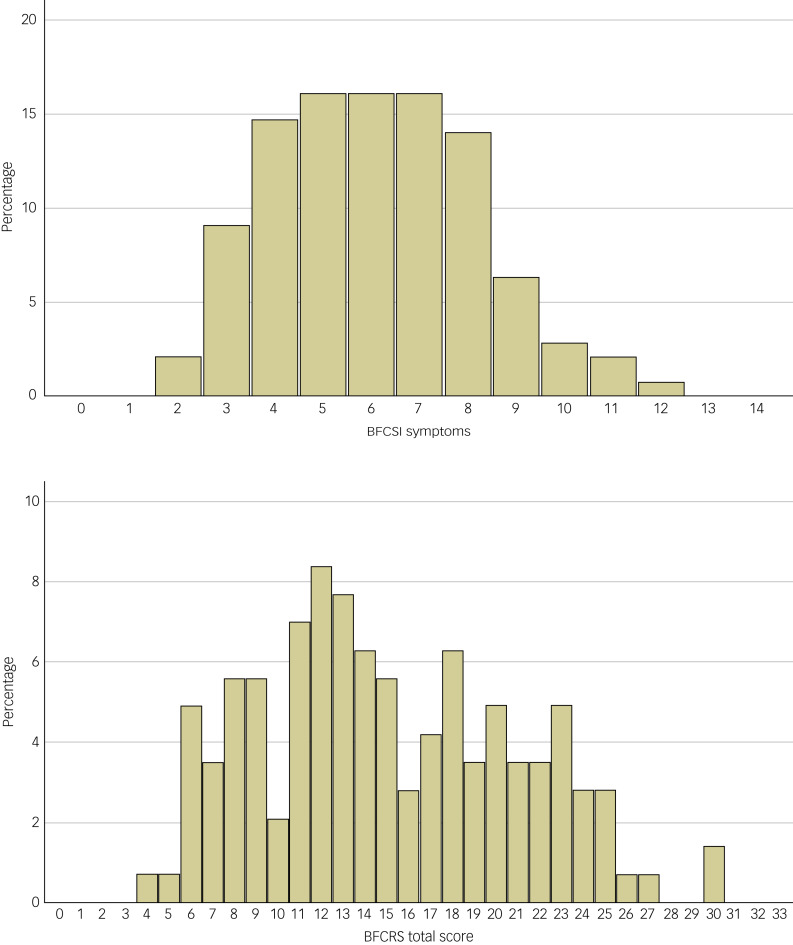
Histogram of the number of BFCSI signs (top) and BFCRS total score (bottom) among 143 paediatric patients with catatonia. BFCRS, Bush Francis Catatonia Rating Scale; BFCSI, Bush Francis Catatonia Screening Item.

**Fig. 2:**
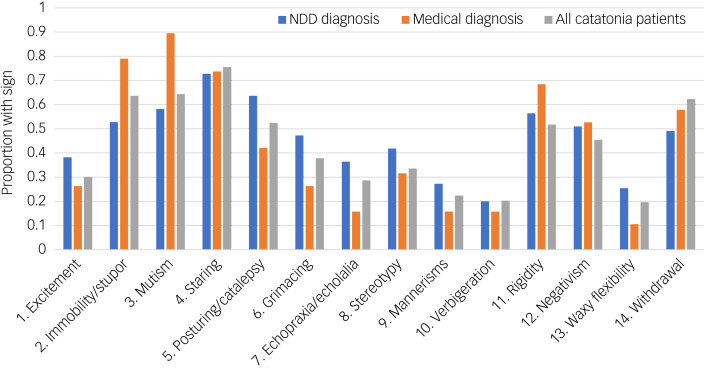
Proportion of paediatric patients with catatonia presenting with each catatonic sign on the BFCSI. Shown are all patients with catatonia (grey), those with a neurodevelopmental disorder (blue) and those with a medical diagnosis (orange). BFSCI, Bush Francis Catatonia Screening Item; NDD, neurodevelopmental disorder.

Paediatric patients diagnosed with catatonia demonstrated a median of six signs (IQR: 4–8) and mean of 6.0 ± 2.1 on the BFCSI (Fig. 1, top; see Supplementary Table 1 available at https://doi.org/10.1192/bjo.2024.61). Among individual signs, staring was most frequently present, with 75.5% of children with catatonia demonstrating this feature (Supplementary Table 2). Patients with neurodevelopmental disorders (*n* = 55) had a median of six signs (IQR: 4–8) and a mean of 6.4 ± 2.3 signs on the BFCSI, which was not significantly different from patients without neurodevelopmental disorder diagnoses (Mann–Whitney *U-*test *P* = 0.182). Patients with a medical diagnosis underlying catatonia (*n* = 19) had a median of seven signs (IQR: 4–8) and a mean of 6.1 ± 2.1 signs on the BFCSI, which was not significantly different from those without a medical diagnosis (Mann–Whitney *U*-test *P* = 0.971). The relative proportion of all patients, of patients with neurodevelopmental disorders and of patients with medical diagnoses displaying each catatonic sign on the BFCSI is given in Fig. 2.

### Catatonia severity

Among the 23 items of the BFCRS, six were present in >50% of patients diagnosed with catatonia (staring, mutism, immobility/stupor, withdrawal, posturing/catalepsy, rigidity), and four were present in <20% of cases (waxy flexibility, mitgehen, gegenhalten, grasp reflex) (Table 3). The median BFCRS score was 14 (IQR: 11–20), with a mean of 15.0 ± 5.9 (Fig. 1, bottom). Overall, lower severity scores were more frequently reported than higher severity scores. Among individual signs, immobility/stupor (51.0%) and staring (49.7%) were most commonly recorded at severity of 1 (‘occasional’), withdrawal (36.4%) and staring (23.1%) were most commonly recorded at a severity of 2 (‘frequent’), and perseveration (35.0%) and ambitendency (32.9%) were most commonly recorded at a severity of 3. Corresponding severity scores for paediatric patients with catatonia with a diagnosis of a neurodevelopmental disorder (*n* = 55) and with a diagnosis of a medical illness (*n* = 19) are listed in Supplementary Tables 3 and 4. Among those with neurodevelopmental disorders, seven signs were present in >50% of patients (staring, posturing, mutism, rigidity, impulsivity, immobility, negativism), whereas for those with medical diagnoses, eight signs were present for >50% of individuals (mutism, immobility, staring, rigidity, impulsivity, withdrawal, negativism, autonomic abnormality).

**Table 3:** Prevalence and severity of catatonic signs from the Bush Francis Catatonia Rating Scale (BFCRS) among paediatric patients diagnosed with catatonia (*n* = 143)

BFCRS item	0, e.g. ‘absent’		1, e.g. ‘occasional’		2, e.g. ‘frequent’		3, e.g. ‘constant’		Present (>0)	
	*n*	%	*n*	%	*n*	%	*n*	%	*n*	%
1. Excitement	100	69.9	28	19.6	12	8.4	3	2.1	43	30.1
2. Immobility/stupor	52	36.4	73	51.0	15	10.5	3	2.1	91	63.6
3. Mutism	51	35.7	42	29.4	25	17.5	25	17.5	92	64.3
4. Staring	35	24.5	71	49.7	33	23.1	4	2.8	108	75.5
5. Posturing/catalepsy	68	47.6	46	32.2	22	15.4	7	4.9	75	52.4
6. Grimacing	89	62.2	36	25.2	13	9.1	5	3.5	54	37.8
7. Echopraxia/echolalia	102	71.3	27	18.9	13	9.1	1	0.7	41	28.7
8. Stereotypy	95	66.4	27	18.9	19	13.3	2	1.4	48	33.6
9. Mannerisms	111	77.6	22	15.4	9	6.3	1	0.7	32	22.4
10. Verbigeration	114	79.7	20	14.0	9	6.3	0	0.0	29	20.3
11. Rigidity	69	48.3	49	34.3	21	14.7	4	2.8	74	51.7
12. Negativism	78	54.5	40	28.0	18	12.6	7	4.9	65	45.5
13. Waxy flexibility	115	80.4					28	19.6	28	19.6
14. Withdrawal	54	37.8	27	18.9	52	36.4	10	7.0	89	62.2
15. Impulsivity	82	57.3	28	19.6	27	18.9	6	4.2	61	42.7
16. Automatic obedience	94	65.7	23	16.1	18	12.6	8	5.6	49	34.3
17. Mitgehen	117	81.8					26	18.2	26	18.2
18. Gegenhalten	122	85.3					21	14.7	21	14.7
19. Ambitendency	96	67.1					47	32.9	47	32.9
20. Grasp reflex	129	90.2					14	9.8	14	9.8
21. Perseveration	93	65.0					50	35.0	50	35.0
22. Combativeness	111	77.6	14	9.8	12	8.4	6	4.2	32	22.4
23. Autonomic abnormality	75	52.4	39	27.3	26	18.2	3	2.1	68	47.6

To compare demographic, clinical and diagnostic features associated with catatonia severity among paediatric patients diagnosed with catatonia, the total BFCRS score was modelled with a generalised linear model, with age, gender, study site and primary diagnosis associated with catatonia (medical, psychotic disorder, mood disorder, neurodevelopmental disorder, unspecified catatonia) as independent variables (Table 4). In this model, compared with those with a diagnosis of unspecified catatonia, patients with a neurodevelopmental disorder as the primary diagnosis associated with catatonia had a higher overall catatonia severity (*β* = 4.75, 95% CI 1.50–8.01; *P* < 0.01), whereas other factors were not significantly associated with catatonia severity.

**Table 4:** Generalised linear model of total Bush Francis Catatonia Rating Scale severity score, with age, gender, study site and primary diagnosis as independent variables

Parameter	*β*	95% CI		*P*-value
		Lower bound	Upper bound	
Age	0.11	−0.19	0.40	0.49
Gender (male)	−0.61	−2.48	1.26	0.52
Study site (site 1)	−2.29	−4.59	0.01	0.05
Diagnosis				
Medical	2.25	−1.45	5.95	0.23
Psychotic disorder	1.50	−1.82	4.82	0.38
Mood disorder	1.18	−2.55	4.91	0.54
Neurodevelopmental disorder	4.75	1.50	8.01	<0.01
Unspecified catatonia	1	Reference	Reference	Reference

## Discussion

This sample of 143 paediatric patients across two large healthcare systems characterises the number, type and severity of catatonic signs according to the BFCRS, in paediatric patients diagnosed with catatonia. Patients in this sample displayed a mean of 6.0 ± 2.1 signs of catatonia on the BFCSI, with a mean BFCRS severity score of 15.0 ± 5.9. To put these findings into context, the largest study utilising the BFCRS in adult patients from a mixed population of psychiatric and medical in-patients found a comparable mean BFCRS score of 14.7 ± 7.8 among 232 individuals.^6^ A further study of 225 adult psychiatric patients with chronic catatonic schizophrenia found a mean BFCRS score of 4.4 ± 4.0, placing the mean paediatric patient in this study among the most severe 0.5% of signs in that study (*Z* score of 2.65).^21^ In contrast, in a prospective cohort of 88 psychiatric paediatric patients who were admitted to hospital and assessed with the Pediatric Catatonia Rating Scale (PCRS) – a 20-item scale derived from the BFCRS and intended specifically for use in children – the mean score was 21.87 ± 7.5,^22^ substantially higher than that reported here.

These differences in catatonia scoring across various cohorts highlight ongoing uncertainty as to optimal catatonia diagnostic criteria, as well as likely variability in catatonia presentation among treatment settings, diagnoses and clinical populations. There are likely distinctions in the clinical features observed in psychiatric samples, those with medical diagnoses, patients of different ages and chronic versus acute catatonia. For example, efforts to characterise catatonic signs specific to ASD have been recently undertaken in meta-analytic research.^23^ Vaquerizo-Serrano and colleagues^23^ found that the most common signs of catatonia in ASD were new-onset speech impairment, negativism and aggression. They also highlighted the significant degree of symptom overlap between signs of catatonia and baseline features of ASD, emphasising the need for clinicians to closely document baseline symptoms of ASD and evaluate for core signs of catatonia that are not present in ASD alone.^23^

ASD and other neurodevelopmental disorders may present with features such as impaired verbal ability, cognitive impairment or impulsivity, which may result in higher baseline BFCRS scores.^23,24^ This overlap between catatonic signs and baseline signs of other disorders may partially explain the higher BFCRS score seen among individuals in this sample with comorbid neurodevelopmental disorders, even after controlling for other demographic features within our generalised linear model. Thus, further research should continue to strive for large sample sizes across multiple sites; include patients of diverse populations, including populations with baseline neurodiversity; and continue to explore diagnostic features that are explicit to catatonia. The utility of alternative catatonia scales designed for specific populations, such as the PCRS for children^22^ and Kanner Catatonia Rating Scale for neurodiverse individuals, should remain an area of active research, particularly given the diversity of catatonia presentations.^25^

Overall, a greater understanding of catatonia across populations is of clinical significance. Misdiagnosis or delayed identification of catatonia may result in progression to malignant catatonia, a condition associated with autonomic instability and mortality rates as high as 10–20% if left untreated.^26^ Furthermore, paediatric catatonia has been associated with a greater than 60-fold higher risk of death than the general population.^19^ Our data, along with existing literature, indicate a high prevalence of medical illnesses associated with paediatric catatonia,^11,22,27,28^ which underscores the necessity of a robust medical examination when catatonia is first diagnosed, as addressing the potential underlying causes is a key component of catatonia clinical care. In some instances, a diagnosis underlying catatonia may itself be treatable (e.g. autoimmune encephalitis with immunomodulatory therapy), whereas in other cases the underlying disorder is not expected to be fundamentally modifiable (e.g. ASD). Encouragingly, research has shown significant clinical responses to benzodiazepines, electroconvulsive therapy (ECT) and adjunctive pharmacologic treatment in children and adults of neurodiverse backgrounds regardless of the underlying disorder associated with catatonia.^29–32^ Restricted and delayed access to ECT in younger patients, however, further highlights the need for quickly identifying catatonia in children and adolescents, because they face barriers to accessing ECT in cases where it may be required.^33–36^

Strengths of this study include a large sample size, making it the largest single sample of paediatric patients diagnosed with catatonia that has been reported. Additionally, the multicentre nature of the study and the inclusion of medical and psychiatric in-patients and out-patients enhances the generalisability of the findings. Limitations of the study derive from the retrospective nature of the cohort generation. To be included in the sample, patients needed to have a documented BFCRS score as well as a clinical diagnosis of catatonia. Although the BFCRS is the standard catatonia assessment in both healthcare systems, patients are not systematically assessed for catatonia signs; therefore, if the treating provider did not appropriately assess for catatonia, the patient would erroneously be recorded as a non-case. It is conceivable that the higher average BFCRS score seen in this cohort may reflect a systematic failure to diagnose less-severe cases of catatonia, thereby skewing the sample toward cases of greater severity. Furthermore, the BFCRS itself is subject to interrater inconsistency, with many catatonic signs misidentified or overidentified by treating providers,^37^ and so results here may be biased based on inaccurate assessment. Reassuringly, overall BFCRS severity did not differ between the two study sites in an adjusted model. Moreover, the BFCRS is validated for adults, but not for children, which should be considered when comparing paediatric and adult data. Additionally, findings in this study of two academic healthcare systems may not directly translate to other treatment settings or populations of different sociodemographic characteristics. As both healthcare systems are paediatric referral centres for broad geographic regions, we cannot assess how representative the clinical population explored here is of broader referral regions. Additionally, this report is based on clinical features observed at the initial diagnosis of catatonia. As catatonic signs can fluctuate over an episode of care, a single cross-sectional analysis may not accurately reflect the clinical features of catatonia present in an individual at other times during the episode of care. Further research should characterise the longitudinal course of catatonia in paediatric patients. Finally, as only cases with a clinical diagnosis of catatonia are included, we are unable to assess for the overall rate of catatonia among paediatric patients because the overall number of non-catatonic patients cannot be determined.

In conclusion, among 143 patients aged 18 years and under in two large healthcare systems with a clinical diagnosis of catatonia on the BFCRS, individuals displayed a mean of 6.0 ± 2.1 signs of catatonia on the BFCSI, with a mean BFCRS severity score of 15.0 ± 5.9. Greater symptom burden was present in individuals with diagnosed neurodevelopmental disorders relative to neurotypical children, although this elevation may be secondary to baseline signs of a neurodevelopmental diagnosis. Staring, mutism and immobility/stupor were the most common catatonic signs among all patients. These results help clarify clinical features of catatonia in youth, and highlight an ongoing need for research into optimal catatonia diagnostic criteria across various populations.

## Supporting information

Smith et al. supplementary material Smith et al. supplementary material

## Data Availability

The data that support the findings of this study are available on request from the corresponding author, J.R.S. The data are not publicly available due to privacy restrictions.
