# Supplementary material for: Diagnostic features of paediatric catatonia: multisite retrospective cohort study
Source: BJPsych Open. 2024 Apr 30;10(3):e96. doi: 10.1192/bjo.2024.61 (PMC11060083; doi:10.1192/bjo.2024.61)
Supplement: Smith et al. supplementary material [file S2056472424000619sup001.docx]

**Supplemental information for:**

**Diagnostic Features of Pediatric Catatonia: a Multisite Retrospective Cohort Study**

Joshua R. Smith, MD^1,2^

Tasia York, MD^1^

Isaac Baldwin, MD^3^

Catherine Fuchs, MD^1^

Gregory Fricchione, MD^4,5^

James Luccarelli, MD, DPhil^4,5^

Author Affiliations:

^1^Division of Child and Adolescent Psychiatry, Department of Psychiatry and Behavioral Sciences; Vanderbilt University Medical Center at Village of Vanderbilt, 1500 21st Avenue South, Suite 2200, Nashville, Tennessee, 37212

^2^Vanderbilt Kennedy Center, Vanderbilt University; 110 Magnolia Circle, Nashville, TN, 37203

^3^Division of General Psychiatry, Department of Psychiatry and Behavioral Sciences; Vanderbilt University Medical Center, 1601 23^rd^ Ave South, Nashville, Tennessee, 37212.

^4^Harvard Medical School, Boston, MA, USA

^5^Department of Psychiatry, Massachusetts General Hospital, Boston, MA, USA

| BFCSI Signs | |
| --- | --- |
| Median, IQR | 6 (4 to 8) |
| Mean ± SD | 6.0 ± 2.1 |
| BFCRS Signs | |
| Median, IQR | 9 (6 to 11) |
| Mean ± SD | 8.7 ± 3.0 |
| BFCRS Score | |
| Median, IQR | 14 (11 to 20) |
| Mean ± SD | 15.0 ± 5.9 |

Table S1: Mean and median signs on the BFCSI and BFCRS and mean and median BFCRS score among all pediatric catatonia patients (N=143).

| BFCRS Feature | N | % |
| --- | --- | --- |
| Staring: | 108 | 75.5% |
| Mutism: | 92 | 64.3% |
| Immobility/stupor: | 91 | 63.6% |
| Withdrawal: | 89 | 62.2% |
| Posturing/catalepsy: | 75 | 52.4% |
| Rigidity: | 74 | 51.7% |
| Autonomic abnormality: | 68 | 47.6% |
| Negativism: | 65 | 45.5% |
| Impulsivity: | 61 | 42.7% |
| Grimacing: | 54 | 37.8% |
| Perseveration: | 50 | 35.0% |
| Automatic obedience: | 49 | 34.3% |
| Stereotypy: | 48 | 33.6% |
| Ambitendency: | 47 | 32.9% |
| Excitement | 43 | 30.1% |
| Echopraxia/echolalia: | 41 | 28.7% |
| Mannerisms: | 32 | 22.4% |
| Combativeness: | 32 | 22.4% |
| Verbigeration: | 29 | 20.3% |
| Waxy flexibility: | 28 | 19.6% |
| Mitgehen: | 26 | 18.2% |
| Gegenhalten: | 21 | 14.7% |
| Grasp reflex: | 14 | 9.8% |

Table S2: BFCRS items ranked by overall prevalence of severity >0 among all pediatric patients with catatonia (N=143).

| BFCRS Feature | 0 e.g. "absent" | | 1 e.g. "occasional" | | 2 e.g. "frequent" | | 3 e.g. "constant" | | Present (>0) | |
| --- | --- | --- | --- | --- | --- | --- | --- | --- | --- | --- |
|  | N | % | N | % | N | % | N | % | N | % |
| 1. Excitement | 34 | 61.8% | 13 | 23.6% | 6 | 10.9% | 2 | 3.6% | 21 | 38.2% |
| 2. Immobility/stupor: | 26 | 47.3% | 25 | 45.5% | 3 | 5.5% | 1 | 1.8% | 29 | 52.7% |
| 3. Mutism: | 23 | 41.8% | 14 | 25.5% | 8 | 14.5% | 10 | 18.2% | 32 | 58.2% |
| 4. Staring: | 15 | 27.3% | 29 | 52.7% | 11 | 20.0% | 0 | 0.0% | 40 | 72.7% |
| 5. Posturing/catalepsy: | 20 | 36.4% | 21 | 38.2% | 10 | 18.2% | 4 | 7.3% | 35 | 63.6% |
| 6. Grimacing: | 29 | 52.7% | 15 | 27.3% | 8 | 14.5% | 3 | 5.5% | 26 | 47.3% |
| 7. Echopraxia/echolalia: | 35 | 63.6% | 12 | 21.8% | 7 | 12.7% | 1 | 1.8% | 20 | 36.4% |
| 8. Stereotypy: | 32 | 58.2% | 13 | 23.6% | 9 | 16.4% | 1 | 1.8% | 23 | 41.8% |
| 9. Mannerisms: | 40 | 72.7% | 11 | 20.0% | 4 | 7.3% | 0 | 0.0% | 15 | 27.3% |
| 10. Verbigeration: | 44 | 80.0% | 7 | 12.7% | 4 | 7.3% | 0 | 0.0% | 11 | 20.0% |
| 11. Rigidity: | 24 | 43.6% | 20 | 36.4% | 10 | 18.2% | 1 | 1.8% | 31 | 56.4% |
| 12. Negativism: | 27 | 49.1% | 16 | 29.1% | 9 | 16.4% | 3 | 5.5% | 28 | 50.9% |
| 13. Waxy flexibility: | 41 | 74.5% |  |  |  |  | 14 | 25.5% | 14 | 25.5% |
| 14. Withdrawal: | 28 | 50.9% | 13 | 23.6% | 12 | 21.8% | 2 | 3.6% | 27 | 49.1% |
| 15. Impulsivity: | 25 | 45.5% | 10 | 18.2% | 16 | 29.1% | 4 | 7.3% | 30 | 54.5% |
| 16. Automatic obedience: | 31 | 56.4% | 10 | 18.2% | 11 | 20.0% | 3 | 5.5% | 24 | 43.6% |
| 17. Mitgehen: | 43 | 78.2% |  |  |  |  | 12 | 21.8% | 12 | 21.8% |
| 18. Gegenhalten: | 47 | 85.5% |  |  |  |  | 8 | 14.5% | 8 | 14.5% |
| 19. Ambitendency: | 33 | 60.0% |  |  |  |  | 22 | 40.0% | 22 | 40.0% |
| 20. Grasp reflex: | 50 | 90.9% |  |  |  |  | 5 | 9.1% | 5 | 9.1% |
| 21. Perseveration: | 34 | 61.8% |  |  |  |  | 21 | 38.2% | 21 | 38.2% |
| 22. Combativeness: | 35 | 63.6% | 7 | 12.7% | 8 | 14.5% | 5 | 9.1% | 20 | 36.4% |
| 23. Autonomic abnormality: | 30 | 54.5% | 15 | 27.3% | 10 | 18.2% | 0 | 0.0% | 25 | 45.5% |
|  |  |  |  |  |  |  |  |  |  |  |

Table S3: Prevalence and severity of catatonic signs from the BFCRS among pediatric catatonia patients with neurodevelopmental disorder diagnoses (N=55).

| BFCRS Feature | 0 e.g. "absent" | | 1 e.g. "occasional" | | 2 e.g. "frequent" | | 3 e.g. "constant" | | Present (>0) | |
| --- | --- | --- | --- | --- | --- | --- | --- | --- | --- | --- |
|  | N | % | N | % | N | % | N | % | N | % |
| 1. Excitement | 14 | 73.7% | 4 | 21.1% | 1 | 5.3% | 0 | 0.0% | 5 | 26.3% |
| 2. Immobility/stupor: | 4 | 21.1% | 9 | 47.4% | 5 | 26.3% | 1 | 5.3% | 15 | 78.9% |
| 3. Mutism: | 2 | 10.5% | 5 | 26.3% | 6 | 31.6% | 6 | 31.6% | 17 | 89.5% |
| 4. Staring: | 5 | 26.3% | 8 | 42.1% | 5 | 26.3% | 1 | 5.3% | 14 | 73.7% |
| 5. Posturing/catalepsy: | 11 | 57.9% | 7 | 36.8% | 1 | 5.3% | 0 | 0.0% | 8 | 42.1% |
| 6. Grimacing: | 14 | 73.7% | 4 | 21.1% | 0 | 0.0% | 1 | 5.3% | 5 | 26.3% |
| 7. Echopraxia/echolalia: | 16 | 84.2% | 2 | 10.5% | 1 | 5.3% | 0 | 0.0% | 3 | 15.8% |
| 8. Stereotypy: | 13 | 68.4% | 2 | 10.5% | 3 | 15.8% | 1 | 5.3% | 6 | 31.6% |
| 9. Mannerisms: | 16 | 84.2% | 3 | 15.8% | 0 | 0.0% | 0 | 0.0% | 3 | 15.8% |
| 10. Verbigeration: | 16 | 84.2% | 2 | 10.5% | 1 | 5.3% | 0 | 0.0% | 3 | 15.8% |
| 11. Rigidity: | 6 | 31.6% | 9 | 47.4% | 4 | 21.1% | 0 | 0.0% | 13 | 68.4% |
| 12. Negativism: | 9 | 47.4% | 6 | 31.6% | 3 | 15.8% | 1 | 5.3% | 10 | 52.6% |
| 13. Waxy flexibility: | 17 | 89.5% |  |  |  |  | 2 | 10.5% | 2 | 10.5% |
| 14. Withdrawal: | 8 | 42.1% | 1 | 5.3% | 9 | 47.4% | 1 | 5.3% | 11 | 57.9% |
| 15. Impulsivity: | 8 | 42.1% | 7 | 36.8% | 3 | 15.8% | 1 | 5.3% | 11 | 57.9% |
| 16. Automatic obedience: | 13 | 68.4% | 3 | 15.8% | 2 | 10.5% | 1 | 5.3% | 6 | 31.6% |
| 17. Mitgehen: | 15 | 78.9% |  |  |  |  | 4 | 21.1% | 4 | 21.1% |
| 18. Gegenhalten: | 15 | 78.9% |  |  |  |  | 4 | 21.1% | 4 | 21.1% |
| 19. Ambitendency: | 16 | 84.2% |  |  |  |  | 3 | 15.8% | 3 | 15.8% |
| 20. Grasp reflex: | 17 | 89.5% |  |  |  |  | 2 | 10.5% | 2 | 10.5% |
| 21. Perseveration: | 13 | 68.4% |  |  |  |  | 6 | 31.6% | 6 | 31.6% |
| 22. Combativeness: | 15 | 78.9% | 4 | 21.1% | 0 | 0.0% | 0 | 0.0% | 4 | 21.1% |
| 23. Autonomic abnormality: | 9 | 47.4% | 3 | 15.8% | 7 | 36.8% | 0 | 0.0% | 10 | 52.6% |

Table S4: Prevalence and severity of catatonic signs from the BFCRS among pediatric catatonia patients with a medical diagnosis associated with catatonia (N=19)
